# Psychological resilience moderates influence of depression on sleep dysfunction of people living with diabetes

**DOI:** 10.1007/s40200-019-00436-9

**Published:** 2019-10-04

**Authors:** Ajele Kenni Wojujutari, Oladejo Teslim Alabi, Idehen Egbeware Emmanuel

**Affiliations:** 1 Department of Psychology, Obafemi Awolowo University, Osun State, Ile-Ife, Nigeria; 2 Department of Mental Health, Federal Medical Center, Lokoja, Nigeria

**Keywords:** Psychological resilience, Depression, Sleep dysfunction and diabetes

## Abstract

**Objectives:**

The study examined the influence of depression on sleep dysfunction in people living with diabetes mellitus and investigated the moderating role of psychological resilience on the influence of depression on sleep dysfunction of patients.

**Methods:**

A cross-sectional survey was carried out among 380 (age 25–77 years; mean = 38.6; SD = 6.07) people living with diabetes who are registered patients and were attending the clinic in Department of Endocrinology, Ondo State Specialist Hospital, Akure.

**Results:**

Results showed that depression significantly influence sleep dysfunction of people living with diabetes, β = 0.3991, 95% CI (0.5393, 0.2588), t = 21.5010, *p* < 0.005. Results also showed significant moderating role of psychological resilience on the influence depression on sleep dysfunction of people living with diabetes, β = 0.7805, 95% CI (0.7091, 0.8519), t = 21.5010, p < 0.005.

**Conclusions:**

Sleep dysfunction of individuals living with diabetes as result of their level of depression could be moderated by patient’s level of psychological resilience. Along these lines, the study concludes that experts should focus more on diabetes patient’s psychological resilience adequacy in their management, guidance and modification programs.

## Introduction

Diabetes mellitus is one of the life-threatening chronic illnesses of the twenty-first century. In 2010, about 285 million individuals were reportedly suffering from the disease, and the numbers are anticipated to become twofold by 2030 [7], while the number is expected to surge to roughly 592 million individuals by the year 2035 [18]. World Health Organization [52] reports that, about 422 million persons are living with the disease globally in 2015. Nigeria has the largest predominance of diabetes mellitus in Africa in 2011 [17]. The prevalence of diabetes has been steadily increasing in Nigeria over the past few decades, particularly among the low and middle-income class [35]. The International Diabetes Federation [19] and WHO [52, 53] reported that about 1.56 million cases of diabetes and 40,815 deaths were recorded in Nigeria in 2014.

According to World Health Organization, [54] approximately 382 million people have diabetes mellitus and 350 million have depression worldwide. Roy and Lloyd [43] found that the prevalence rate of depression is more than three times higher in people with type 1 diabetes (12%, range 5.8–43.3% vs. 3.2%, range 2.7–11.4%), and nearly twice as high in people with type 2 diabetes mellitus (T2DM) (19.1%, range 6.5–33% vs. 10.7%, range 3.8–19.4%) when compared to these without diagnoses. Also, women with, and without diabetes are observed to experience a higher prevalence of depression than men. Alonso-Morán et al. [1] reported a prevalence of 9.8% (CI 95% [9.6, 9.9]) women with type 2 diabetes. Furthermore, Rajender et al. [41] reported that one-third of persons with T2DM (32.05%) suffered from major depression.

In recent years, there has been a growing awareness of multiple interrelationships between depression and various physical diseases such as heart disease, cancer and most especially diabetes (Katon, Maj & Sartorious, [21]). Depression is a frequent and serious comorbid condition in diabetes, which may adversely affect their psychological well-being and long-time prognosis. Co-current depression may present peculiar clinical challenges, making both conditions harder to manage either by the patient or health professionals. According to the Fifth edition of Diagnostic and Statistical Manual Disorders (DSM-5; APA 2013) individuals with depression will experience these symptoms most of the day, nearly every day, for at least two weeks such as depressed or irritable mood, loss of interest or pleasure in activities, appetite disturbance, poor sleep quality, retardation, loss of energy difficulty to make decisions, feeling of worthless or excessive guilt and recurrent thoughts of death.

Depressed persons living with diabetes mellitus may be suffering due to several factors such as chronic illness and sleep dysfunction. The complications produced coexistence of depression and diabetes mellitus are not only deteriorating patient’s physical health, but also their emotional instability. Zhang et al. [55] suggested that people living with diabetes experience depression and sleep dysfunction as a result of the prolonged ailment and requirements of self-care.

Depressed persons with diabetes mellitus may be experiencing feelings of sadness, hopelessness, loneliness, abnormal eating and sleeping patterns, and irritability, which are symptoms of depression. Depressed persons with diabetes may also consistently distort their interpretations of their predicament so that they maintain negative views of themselves, the environment, and future. This negative perception of their aliment can also disparage their psychological well-being. These distortions represent deviations from the logical processes of thanking typically used by persons with diabetes may have profound consequence on their psychological well-being. According to WHO [54]; Tiki [46] depression is a common mental disorder that manifests with alongside a depressed mood, loss of interest or pleasure, decreased energy, feelings of guilt or low self-worth, disturbed sleep or appetite, and poor concentration.

Sleep dysfunction is a manifestation of depression which is found to be common among persons with chronic illnesses. Khandelwal et al. [25] found that most individuals living with diabetes mellitus experience sleep dysfunction but that it is more common among individual with type 2 diabetes. Skomro et al. [44] reported that individuals living with diabetes mellitus experience a high level of insomnia, poor sleep quality, excessive daytime sleepiness, and therefore, become higher users of sleep medications. Persons with diabetes mellitus may be experiencing sleep dysfunction because of associated physical complications such as peripheral neuropathy and polyuria [25]. Studies have identified such factors: chronic pain associated peripheral neuropathy, restless legs syndrome, periodic time movements, rapid changes in blood glucose levels during the night leading to hypoglycaemic episodes, nocturia and associated depression, restriction, and excessive sleep and sleep fragmentation [3, 42]. Some studies have revealed that sleep dysfunction is a risk factor for persons with diabetes mellitus especially type2 diabetes [25, 27].

Zhu et al. [56] described sleep dysfunction in individuals with diabetes as a perplexing indication that incorporates disabled sleep quality as well as unusual sleep term. Dysfunctional sleep has been frequently found among diabetes patients [22, 48, 56]. Mirghani and Elbadawi [33] identified excessive daytime sleepiness and depression besetting the health of diabetes mellitus patients. Polo-Kantola et al. [38] found dysfunctional sleep to cross-sectional associate with depression and anxiety symptoms. Similarly, Volkovich et al. [49] reported that emotional distress (depression and anxiety symptoms severity) was associated with subjective sleep dysfunction. Also, Maglione, et al. [30] found a significant relationship between sleep disturbance and depression in a cohort of older women with the diagnosis. Paudel et al. [37] found a significant relationship between sleep disturbance and risk of depression in older men. Similarly, Boa, et al. [4] observed that sleep disturbance influence the risk of the onset of depression in older adults.

According to the *Diagnostic and Statistical Manual of Mental Disorders: Fifth Edition* (DSM-; APA, 2013), sleep dysfunctions are often accompanied by feelings of depression, anxiety, and cognitive changes that should be properly managed, and must be addressed in treatment planning. As indicated in the manual sleep dysfunctions are produced by the prodromal articulation of a scene of psychological maladjustment, enabling the likelihood of early intercession to pre-empt, or to weaken anepisode. Therefore, people living with diabetes mellitus require maximum psychological resilience to adjust to their prolonged self-management of the ailment and the associated sleep dysfunction and depression. Psychological resilience refers to a possession of pressure adapting capacity, that could be an imperative of focus in the treatment in anxiety, depression, and stress responses ([9]; Taylor, [45]).

Psychological resilience is a required psychological resources for individual to face overcome and emerge strengthened or transformed from experiences of misfortune [15]. Psychological resilience has been defined as the process of adapting well in the face of adversity, trauma, tragedy, threats, or significant sources of stress such as family and relationship problems, serious health problems or workplace and financial stressors (APA, [2]). Psychological resilience is a coping mechanism that enables individuals to adapt to the circumstances they encounter emotionally, physically and economically. Fergus and Zimmerman [12] described psychological resilience as an individual’s positive adaptation to life threatening challenges. Mariano et al. [31] suggested that psychological resilience is an adapting system that empower individual towards early change and adjustment to ominous circumstances. Hence, persons with diabetes mellitus can learn and develop psychological resilience skills in order to adjust to health challenges. Galli and Vealey [13] postulates that psychological resilience is a fundamental part of the adaptive process, whereby people utilize a scope of adapting procedures to manage a mix of unpalatable feelings and mental struggles.

Previous studies showed that psychological resilience effectively moderates pain, bipolar disorder, and suicidality [8, 32, 34]. In contrast, psychological resilience was not found to be a mediator between emotional dysregulation and Generalised Anxiety Disorder (GAD), however, the relationship between emotion dysregulation and worry was partially mediated by resilience (Webster, [50]). Findings have also showed a significant positive correlation between resilience and subjective well-being among survivors of dengue fever ([20, 24, 40, 47]; Mahmood & Ghaffar, 2014). Grossman’s [14] found that resilience and hardiness moderately to strongly relate to health and well-being outcomes, in the anticipated directions, as well as posed as mediators of positive emotion, and adaptive coping. Psychological resilience was found to be a facilitator of higher levels of optimism, a construct that is known to foster higher levels of psychological well-being [28]. Another study found a significant association between psychological resilience and decreased incidence of stress and anxiety [10]. Mariano et al. [31] found that psychological characteristic resilience moderated the effect of emotional exhaustion on the psychological health of nursing students.

## Objectives of the study

The main objective of the study was to assess sleep dysfunction in people living with diabetes mellitus. The specific objectives were to examine the influence of depression on sleep dysfunction among people living with diabetes mellitus and investigated the moderating role of psychological resilience on the influence depression on sleep dysfunction in people living with diabetes mellitus.

## Methods

### Design

Across-sectional survey design was used in the study. Primary data was collected through the administration of a set of standardised psychological scales on a convenient sample of the study population. Also, patients’ data such as diagnosis, and socio-demographic were collected from their files.

### Participants

The study population consisted of 5721 people living with diabetes who were registered patients in and were attending the endocrinology clinic of the Ondo State Specialists Hospital, Akure, Nigeria. A total of 380 (204 male and 176 female) participants were selected for the study using the convenient sampling technique whereby only patients available on four consecutive clinic days were approached individually and those who consented were included in the study.

The sample consisted of 151(39.7%) person with T1DM and 229(60.3%) and person with T2DM participants in study (presented in Table 1). The majority 210 (55.3%) of the respondents were receiving Insulin injection type of management, while the remaining 170 (44.7%) of the respondents were receiving oral drug type of management (presented in Table 1). The age of the 380 respondents ranged from 25 to 77 years, *mean* = 38.6, and SD = 6.07.

**Table 1:** Summary of Analysis of Respondents Clinical Characteristics

Variables	Groups	Frequency	Percentages (%)
Type of diabetes	Type 1	151	39.7
	Type 2	229	60.3
Type of management	Insulin injection	210	55.3
	oral drugs	170	44.7

### Instruments

#### Medical outcomes study (MOS) sleep scale

The Medical Outcomes Study (MOS) Sleep Scale by Hays and Stewart [16] was used to assess the diabetes mellitus patients sleep dysfunction. The MOS Sleep Scale is a 12-item measure assessing six domains of sleep: 1) sleep disturbance (e.g., the ability to fall and stay asleep), 2) sleep adequacy (e.g., sleeping enough to feel rested and restored), 3) sleep quantity (e.g., the number of hours slept), 4) somnolence (e.g., daytime sleepiness), 5) snoring, and 6) shortness of breath or headache. The MOS Sleep Scale uses a variety of response sets. Item 1 queries how long it takes the patient/respondents to fall asleep. Response options are blocked into “0–15 min,” “16–30 min,” “31–45 min,” “46–60 min,” and “more than 60 minutes.” Item 2 queries how many hours of sleep were obtained on average over the past four weeks. This is an open-ended question ranging between 0 and 24 h. The remaining 10 items use a 6-point Likert-type scale based upon the following values and anchors (1 = all of the time, 2 = most of the time, 3 = a good bit of the time, 4 = some of the time, 5 = a little of the time, and 6 = none of the time). The MOS sleep scoring range from 0 to 100 in which higher scores indicating greater severity of sleep disturbance.

The MOS has constantly demonstrated good internal consistency with Cronbach’s that alpha ranging between 0.64–0.84 for the MOS subscales. In restless legs syndrome, all scales exceeded Cronbach’s alpha of 0.70 with the exception of somnolence (0.66). All multi-item scales (i.e., sleep disturbance, sleep adequacy, somnolence, and summary indices) exceeded 0.70. Kim et al., [26] found that internal consistency of reliability for the MOS sleep scale was 0.80 as measured by Cronbach’s alpha among patients with painful diabetic peripheral neuropathy in Korea.

#### Beck depression inventory (BDI-II)

Beck Depression Inventory by Beck, Ward, Mendelson, Mock and Erbaugh [6], was used to measure depression in the study. The BDI-II is the most widely used self-report measure of depression (Dozois, [39]). It consists of 21 items. Each item is rated on a 4-point scale ranging from 0 to 3, and scores are totalled and range from 0 to 63. The BDI-II can be administered to groups or individuals in 5–10 min. Scores of 0–13 indicate “minimal” depression, 14–19 indicate “mild” depression, 20–28 indicates “moderate” depression, and 29–63 indicate “severe” depression [5].

The intended population for the BDI-II consists of individuals who are 13–80 years of age. The psychometric properties of the BDI-II are sound. Specifically, the BDI-II has a high internal consistency of 0.91, retest-reliability of 0.93, and convergent validity with the Hamilton Psychiatric Rating Scale for Depression (r = 0.71) (Beck, Steer, Ball, & Ranieri, [5]). Deassalegn, Yemataw and Atinkut [11] found Cronbach’s alpha 0.91 for the Beck Depression Inventory (BDI-II) among diabetes patients.

#### Conner-Davison resilience scale (CD-RISC)

The Conner-Davison Resilience Scale (CD-RISC) developed by Connor and Davidson [9], was used to assess the patients psychological resilience. The CD-RISC comprise of 25-items. Each item is rated on a 5-points Likert scale ranging from 0 = not true at all time to 4 = true nearly all the time. A high score indicates greater psychological resilience. The CD-RISC constantly demonstrated good internal consistency of Cronbach’s alpha 0.87 [9] as well as excellent construct validity as demonstrated by a positive correlation with the Kobasa Hardiness scale (r = 0.83).

### Procedure

Ethical clearance was obtained from the Research Ethics Committee of the State Specialist Hospital Akure. Participants gave informed consent before they were enrolled in the study. The researchers were introduced by the consultant to the nurses, patients and other members of the staff in the department of endocrinology. This was to ensure the maximum cooperation of the nurses and other staff of the hospital in enlisting patients who meet the criteria for inclusion in the study. The study questionnaire was administered individually by the researchers on clinic days (Mondays and Fridays) within the hospital premises. The participants filled questionnaires during their waiting time to see their doctors for consultations. Although waiting times were often long, some of the participants completed their questionnaires after consultations.

In order to ensure an anonymous process while collecting the data, a drop box was placed in the waiting room. Participants were informed about the drop box and the importance of anonymity, however, some participants felt uncomfortable with this instruction and asked the researcher if they could submit the questionnaire personally. The researchers retrieved the questionnaires from the participants who felt uncomfortable using the drop box and also packed those ones that were dropped in the drop box at the end of each clinic day. A researcher was always present in the waiting room during the data collection process, in order to give clarifications and also provide answers to all the participants’ questions about the study.

### Data analysis

The data were analysed using regression, independent sample t-test, and MANOVA. The analyses were carried out with sub-programs of the IBM/SPSS Version 22.0.

## Results

The result presented Table 2 showed that the MOS-Sleep Scale had (mean = 26.83, SD = 5.87), BDI-II had (mean = 42.15, SD = 7.78), and CD-RISC had (=45.09, SD = 25.26). The results presented Table 3 further showed that there was a positive significant relationship between DBI-II and CD-RISC (r = 0.192). The results further showed a positive significant relationship between the MOS-Sleep Scale and CD-RISC (r = 0.794).

**Table 2:** Summary of descriptive analysis of measures (*N* = 380)

Variables	Mean	SD
MOS-Sleep Scale	26.83	5.87
BDI-II	42.15	7.78
CD-RISC	45.09	25.26

**Table 3:** Summary of multiple pearson correlation between measures (*N* = 380)

Variables	Mean(SD)	MOS-Sleep Scale	DBI-II	CD-RISC
MOS-Sleep Scale	26.83(5.87)	1		
DBI-II	42.15(7.78)	0.794^**^	1	
CD-RISC	45.09(25.26)	0.174^**^	0.192^**^	1

The results presented in Table 4 showed that people living with Type 2 diabetes experience a slight but significant level of depression **(**mean **=** 44.61, *SD* = 10.81) than Type 1 diabetes patients (mean = 42.92, *SD* = 11.58, *t* = 1.428, *p* < 0.05). Also, the results showed that an individual receiving insulin injection experience a slight but significant depression **(**mean **=** 44.19, *SD* = 10.63) than individuals using oral drugs management (mean **=** 42.85, *SD* = 12.05, t = 1.158, p < 0.05). Furthermore, the results showed there was no statistically significant difference people living with Type1 diabetes psychological resilience (mean **=** 47.32, *SD* = 24.34) and people living with Type 2 diabetes (mean = 43.63, *SD* = 25.80, t = 1.393, *p* > 0.05).

**Table 4:** Summary of analysis of sample independent t-test on depression, psychological resilience and sleep dysfunction type of diabetes and type of management

Variables	Groups	***n***	Mean(SD)	df	t	P
Depression	Type1 Diabetes	151	44.61(10.81)	378	1.428	0.000
	Type2 Diabetes	229	42.92(11.58)			
	Insulin injection	210	44.19(10.63)	378	1.158	0.003
	Oral drugs	170	42.85(12.05)			
Psychological resilience	Type1 Diabetes	151	47.32(24.34)	378	1.393	0.244
	Type 2 Diabetes	229	43.63(25.80)			
	Insulin injection	210	43.28(24.36)	378	1.561	0.078
	Oral drugs	170	47.34(26.23)			
Sleep dysfunction	Type1 Diabetes	151	25.64(5.92)	378	4.974	0.000
	Type2 Diabetes	229	28.62(5.32)			
	Insulin injection	210	27.61(5.68)	378	2.919	0.004
	Oral drugs	170	25.85(5.98)			

The results also revealed that there was no statistically significant difference individual on oral drugs level of management psychological resilience **(**mean **=** 47.34, *SD* = 26.23) and insulin injection **(**mean **=** 43.28, *SD* = 24.36, t = 1.561, p > 0.05). Lastly, the results showed that people living with Type 2 diabetes experience more sleep dysfunction **(**mean **=** 28.62, *SD* = 5.32) than Type 1 diabetes patients (mean = 25.64, *SD* = 5.92, t = 4.974, *p* < 0.05) and patients receiving insulin injection management **(**mean **=** 27.61, *SD* = 5.68) more sleep dysfunction than those on oral drugs **(**mean **=** 27.61, *SD* = 5.98, t = 2.919, p < 0.05).

The results presented in Table 5 showed that depression had a significant influence on sleep dysfunction in people living with diabetes [β = 0.3991, 95% CI (0.5393, 0.2588), t = 21.5010, *p* < 0.005]. The result also showed that psychological resilience has a significant influence on the depression of people living with diabetes [β = 0.1520, 95% CI (0.104, 0.07), t = 6.1980, p < 0.005]. The results further showed that psychological resilience significantly moderated the influence of depression on sleep dysfunction of people living with diabetes [β = 0.7805, 95% CI (0.7091, 0.8519), t = 21.5010, p < 0.005]. The model explained 41.1% of the total variability of the sleep dysfunction of people living with diabetes (F_3, 376_ 249.69, *P* < 0.05). This indicate that the sleep dysfunction of people living with diabetes and are influenced by depression is moderated by their psychological resilience.

**Table 5:** Summary of regression analysis by psychological resilience as a moderator of the influence of sleep dysfunction on depression (Note: R = 0.641, R^2^ = 0.411, F (3,376) 249.69, P < 0.005)

Variable	Β	SE B	t	P
Constant	38.72 (37.75, 39.69)	0..4913	78.805	0.001
Depression	0.3991 (0.5393, 0.2588)	0.0713	5.5936	0.001
Psychological resilience	0.7805 (0.7091, 0.8519)	0.0363	21.5010	0.001
Psychological resilience*Depression	0.0253 (0.0139, 0.0367)	0.0058	4.3525	0.001

The results presented in Table 6 revealed that psychological resilience had a statistically significant main effect on sleep dysfunction (F_2, 377_ = 65.682, *p* < 0.05). Further examination of this finding with the Scheffee’s post-hoc test presented in Table 7 showed that patients with high level of psychological resilience (mean = 24.71) had less sleep dysfunction than those with low level of psychological resilience (mean = 20.51, MD = 4.19, *p* < 0.05). Those with moderate levels of psychological resilience (mean = 28.97) also had less sleep dysfunction those with low levels of psychological resilience (mean = 20.51, MD = 8.46, *p* < 0.05).

**Table 6:** Summary of multivariate analysis on sleep dysfunction and depression by psychological resilience

Source	Dependent variable	df	SS	MS	F	P
Psychological resilience	Sleep Dysfunction	2	3457.687	1728.844	65.682	.001
	Depression	2	9521.335	4760.668	133.608	.001
Error	Sleep Dysfunction	377	9923.113	26.321		
	Depression	377	13,433.115	35.632		
Total	Sleep Dysfunction	380	286,312.000			
	Depression	380	698,071.000

**Table 7:** Summary of post hoc tests multiple comparisons of sleep dysfunction and depression by psychological resilience groups

Variables	Gruop_1_	Gruop_2_	M_1_	M_2_	Std. Error	MD	P
Sleep dysfunction	Moderate	Low	28.97	20.51	0.76	8.46	0.01
	High	Low	24.71	20.51	0.87	4.19	0.01
Depression	Moderate	Low	45.73	39.28	1.66	6.44	0.01
	High	Low	40.27	39.28	1.91	0.98	0.01

The results presented in Table 6 also showed that psychological resilience had a statistically significant main effect on depression (F_2, 377_ = 133.608, p < 0.05). The results of post-hoc test presented in Table 7 showed that patients with low levels psychological resilience (mean = 39.28) were prone to more symptoms of depression than those with high levels of psychological resilience (mean = 40.27, MD = 0.89, p < 0.05) and those with moderate levels (mean = 45.73, MD = 8.46, p < 0.05).

## Discussion

The study examined the influence of depression on sleep dysfunction among people living with diabetes. The findings of the study concurs with that of previous studies that found dysfunctional sleep among diabetes patients to be frequent ([22, 48]; Zhu, 2017). The finding also corresponds with that of previous studies which showed that people living with diabetes experience emotional burdens and sleep dysfunction as a result of the illness continuous requirement of self-care [55]. The findings are also in line with that of Boa, et al., [4], who found that sleep disturbance influence the risk of the onset of depression in older adults.

The findings of this also confirmed those of Maglione, et al., [30] who found a significant relationship between sleep disturbance and depression in a cohort of older women. The finding also support the results of Paudel et al. [37] who showed a significant relationship between sleep disturbance and risk of depression in older men. The findings also concur with the previous studies that ascertained that emotional distress (such as depression and anxiety symptoms severity) are associated with subjective sleep dysfunction [49].

The study found that psychological resilience moderated the influence of depression on sleep dysfunction of people living with diabetes. These findings are congruent with the previous studies on the moderating role of resilience between emotional deregulation and generalised anxiety disorder Webster, [50]. The findings are also in agreement with that of Grossman [14] who revealed that resilience and hardiness are both moderately and strongly relate with health and well-being outcomes. The finding is also in line with that of study of Davydov et al. [10] which found a significant association between psychological resilience and decreased incidence of stress and anxiety. The result confirmed the findings of previous studies that found psychological resistance to effectively moderate pain, bipolar disorder, and suicidality [8, 32, 34].

## Limitations of the study

Firstly, the findings of this study appear to be relevant only within its scope and setting. This is because the participants used for the study were relatively small and were drawn from only one hospital in Ondo state. Thus, the generalizability of the findings of this study to other states in Nigeria may be nearly impossible. Secondly, the data collected were based on self-reports which gave room for participants’ bias response to the questions, and may have tendencies of depicting social desirability.

## Directions for future research

Further studies should get responses from other states in Nigeria and also increase the population of subsequent research above the number of respondents used in the study. Qualitative method of data collection such as the use of interview and focus group discussion should be incorporated in further studies by other researchers that would want to consolidate on the findings of this study. Furthermore, other variables that could influence the outcome of this study should be taken into consideration for further studies.

## Conclusion

The study concluded that sleep dysfunction in individuals living with diabetes is impacted by depression could be moderated by psychological resilience. Consequently, specialists should focus more on the promotion and improvement of psychological resilience by adapting systems in diabetes management, treatment, as well as directing and alteration programs.
